# Hippocampal microglial activation triggers a neurotoxic-specific astrocyte response and mediates etomidate-induced long-term synaptic inhibition

**DOI:** 10.1186/s12974-020-01799-0

**Published:** 2020-04-07

**Authors:** Dongliang Li, Mingming Chen, Tao Meng, Jianchun Fei

**Affiliations:** 1 Department of Anesthesiology, Qilu Hospital of Shandong University, Ji’nan, 250012 China; 2 Jiangsu Key Laboratory of Drug Screening, China Pharmaceutical University, Nanjing, 210009 China

**Keywords:** General anesthetics, Inflammation, Synaptic plasticity, IPSC, EPSC, magnetic-activated cell-sorting

## Abstract

**Background:**

Accumulating evidence has highlighted the importance of microglial and astrocyte responses in the pathological development of postoperative cognitive dysfunction (POCD). However, the mechanisms involved are not well understood.

**Methods:**

A perioperative neurocognitive disorders (PND) mouse model was generated by administering etomidate, and cognitive function was assessed using the Morris water maze and novel object recognition tests. Excitatory and inhibitory postsynaptic currents were recorded to analyze neuronal activity. In addition, microglia and astrocytes were isolated by magnetic-activated cell sorting, and genes that were activated in these cells were identified using quantitative polymerase chain reaction.

**Results:**

We observed dramatic cognitive impairment at 1 and 3 weeks after etomidate was administered to 18 month-old mice. Microglia and astrocytes isolated from the hippocampus showed significant microglial activation during the early pathological stage (i.e., 1 week after etomidate injection) and an A1-specific astrocyte response during the late pathological stage (i.e., 3 weeks after etomidate injection). Furthermore, when microglia were eliminated before etomidate was injected, the A1-specific astrocyte activation response was significantly reduced, and cognitive function improved. However, when microglia were eliminated after etomidate application, astrocyte activation and cognitive function were not significantly altered. In addition, activating microglia immediately after a sedative dose of etomidate was injected markedly increased A1-specific astrocyte activation and cognitive dysfunction.

**Conclusions:**

A1-specific astrocyte activation is triggered by activated microglia during the initial pathological stage of PND and induces long-term synaptic inhibition and cognitive deficiencies. These results improve our understanding of how PND develops and may suggest therapeutic targets.

## Background

General anesthetics may induce postoperative cognitive dysfunction (POCD), now progressively recognized as perioperative neurocognitive disorders (PND), which occurs mainly among elderly patients and is characterized by cognitive deficiencies, memory loss, and decreased quality of life [1–5]. Despite excellent research in fiber photometrics, optogenetics, and chemogenetics showing how clinical anesthetics affect the central nervous system and produce side effects [6], the precise mechanisms involved remain elusive.

Current data suggest that all the effects of general anesthetics are derived from synaptic inhibition [7, 8], including forms of inhibition that are essential (e.g., loss of consciousness and immobility during surgery) and forms of inhibition that are undesired (e.g., subsequent cognitive impairment and memory loss). Synaptic inhibition produces transient inhibitory currents and occurs due to dysfunction of ligand-gated ion channels and neurotransmission. This, in turn, is due to a high concentration of γ-aminobutyric acid (GABA) in the synaptic cleft, which activates postsynaptic terminal GABA type A receptor (GABAA-R) channels [9, 10]. In addition, persistent synaptic inhibition, which results in an inhibitory current after the anesthetic has been eliminated, is probably due to complex effects produced by cytokines that are secreted by astrocytes, which express GABAA-Rs on the synaptic surface [10–14]. However, little is known about the initial events that trigger the neurotoxic astrocytic response.

Accumulating evidence has suggested an important role for microglial activation in triggering the neurotoxic astrocytic response (i.e., A1-specific reactivation). For example, activated microglia induced A1-specific astrocytic responses by secreting multiple cytokines (including interleukin [IL]-1α, tumor necrosis factor [TNF]α, and C1q), whereas lipopolysaccharide (LPS) failed to induce such responses in Csf1r knockout mice lacking microglia and impair synaptic plasticity [15]. Furthermore, A1-specific astrocytes activated by microglia have also been detected in neurodegenerative transgenic mice (e.g., 5XFAD, R6/2, and SOD1-G93A mice) and shown to propagate inflammatory effects [16]. In addition, suppressing the activation of microglia and neuroinflammation have been identified as promising approaches for improving cognitive function after general anesthesia [17–19]. Therefore, we suspect that microglial activation is involved in long-term cognitive dysfunction, mainly by triggering neurotoxic astrocytic responses.

To test our hypothesis, we established PND mice using etomidate injections and investigated microglial and astrocyte responses. We observed significant non-specific microglial activation at 1 week and A1-specific astrocyte activation at 3 weeks after etomidate injection. Next, we eliminated the microglia using pexidartinib (PLX3397) and further assessed the astrocytes and cognitive function. The data showed that microglial activation had to be suppressed during the initial pathological stage to prevent A1-specific astrocyte induction and rescue cognitive dysfunction. Finally, we found that activating microglia before a sedative dose of etomidate was administered was sufficient to trigger A1-specific astrocytic responses and impair cognitive function. These data suggest that microglial activation during the early pathological stage induces long-term cognitive dysfunction by triggering A1-specific astrocytic changes and sustained synaptic inhibition.

## Methods

### Animals

In this study, male Thy1-egfp mice purchased from Jackson Labratories (Bar Harbor, ME, USA) were used. All animals were fed in a standard mouse chow and water ad libitum, housed in the animal center of Shandong University with a temperature and humidity-controlled environment, and under a 12:12 light: dark cycle. All procedures were approved by the Administration Committee of Experimental Animals, Shandong Province and Shandong University.

### Anesthesia

According to the data from previous study [20] in establishing the relationship between loss of righting reflex and effective dose (ED_50_ and ED_100_), the anesthetizing and sedative dosage of etomidate were set as 20 mg/kg, i.p. and 8 mg/kg, i.p. respectively in aged (18-month-old) mice. At the same time, aged (18-month-old) mice received equal volume of physiological saline (i.p.) were designated for control. To avoid hypothermia or hypoxia, we monitored the body temperature and heart rate by pulse oximetry sensor (MouseOx, Starr Life Sciences Corp., USA). After general anesthetics, mice were allowed to recovery in a temperature controlled (35 ± 1 °C) acrylic chamber flushed with medical air (30% O_2_, 70% air).

### Behavioral function test

Behavioral function test (12 mice per group) including Morris water maze (MWM) test and novel object recognition (NOR) test were performed according to previous study [21].

In detail, MWM test is divided into three sessions and lasts 8 days (4 trails per day). Before the test, a black plastic pool (100 cm in diameter and 40 cm in height) was filled with water (adding white food color and controlling temperature at 20 ± 1 °C). At day 1 and day 2 (visible platform training), we placed platform 0.5 cm above the water surface and placed mice in the opposite quadrant to platform quadrant. At day 3 to day 7 (hidden platform training), we transfer platform to the opposite quadrant compared to visible platform test and filled water to let water surface 0.5 cm over platform, then mice were placed in the opposite quadrant to platform quadrant. From day 1 to day 7, mice were allowed to swim and find platform for escaping from water in 60 seconds per trail. If mice fail to find platform in 60 seconds, we lead them swimming to the platform by a stick and allow them stay in the platform for 10 seconds. At day 8 (transfer test), we remove platform from pool and placed mice in the opposite quadrant to platform quadrant and allow mice swimming in the pool for 90 seconds. During each trail, swim path of mice were recorded and average swimming speed (m/sec) and time (s) required to reach the platform were calculated by ANY-maze (Global Biotech Inc., NJ, USA).

Novel object recognition test is divided into 3 sessions in an open field (50 cm in length, 50 cm in width, 40 cm in height) and lasts for 3 days. At day 1 (habituation session), mice were placed in the center of open field and allowed to adapt the context for 10 min. At day 2 (acquisition session), mice were placed in the center of open field with two identical objects in it and allowed to recognize objects for 5 min. At day 3 (testing phase), we first replace one of the object with a novel object different in shape and color and then placed mice in the center of open field and allowed them to recognize objects for 5 min. We recorded the whole test by ANY-MAZE software (Global Biotech Inc., NJ, USA) and further analyzed total exploration time (s) and the discrimination index (DI, DI = (time exploring the novel object- time exploring the familiar object)/total exploration time).

### Electrophysiological analysis

The patch clamp whole-cell recording were applied to pyramidal neurons in DG region and EPSC and IPSC were detected and analyzed according to the protocol from previous study [22]. In brief, 350 um-thick brain slices were prepared in pre-cold oxygenated (95% O_2_/ 5% CO_2_ for more than 2 hours) artificial cerebrospinal fluid (high-sucrose version of aCSF: 87mM NaCl, 2.5mM KCl, 7mM MgCl_2_, 1.25mM NaH_2_PO_4_, 25mM NaHCO_3_, and 25mM glucose, 75mM sucrose, pH 7.3). Then, transferred brain slices into a culture chamber and recovered for 2 hours, perfusing the chamber with oxygenated aCSF (120mM NaCl, 3mM KCl, 4mM MgCl_2_, 1mM NaH_2_PO_4_, 26mM NaHCO_3_, and 10mM glucose, pH 7.3) and maintained temperature between 32-34 °C. After that, we finally transferred brain slice into recording chamber (perfused with aCSF and maintained temperature between 32-34 °C) and pyramidal neurons in DG region and EPSC and IPSC were detected by micropipette were filled with working buffer (1mM MgCl2, 0.2mM EGTA, 4mM Mg-ATP, 0.3mM Na-GTP, 125mM Cs- methanesulfonate, 5mM CsCl, 10mM phosphocreatine and 5mM QX314; pH 7.3, 285 mOsm), under an upright microscope equipped with a 40× water-immersion lens (Axioskop 2 Plus, Zeiss). The sEPSC ( -60mV) and sIPSC (0mV) data were obtained by MultiClamp 700B amplifier and 1440A digitizer (Molecular Device, USA). Then, mEPSC ( -60mV) and mIPSC (0mV) data were obtained in the presence of 1 μum TTX (MCE, China).

### Magnetic-activated cell-sorting (MACS)

To assess the response state of microglia and astrocyte in hippocampus, we isolated astrocyte by using magnetic-activated cell-sorting. First, hippocampus were separated from mice and immediately incubated with digestion buffer (300μg/ml DNasel (Sigma-Aldrich, USA) and 1mg/ml papain (MCE, China) at 37 °C for 30 minutes. After that, we centrifuged lyses at 300 *g* for 15 minutes and collected cell pellets for microglia and astrocyte isolation according to the manufacturer’s instructions of CD11b (Microglia) MicroBeads and Glast- MicroBeads kit (Miltenyi Biotech, Germany) respectively.

### Flow cytometry

Flow cytometry was used to confirm the purity of microglia and astrocyte after MACS and the number of microglia in hippocampus after PLX3397 chow. The hippocampus separated from mice were immediately incubated with digestion buffer (300ug/ml DNasel (Sigma-Aldrich, USA) and 1mg/ml papain (MCE, China) at 37 °C for 30 minutes. After that, we centrifuged lyses at 300 g for 15 minutes and collected cell pellets. The cell pellets together with ones obtained after MACS then were suspended with phosphate buffer saline (PBS) buffer and incubated with corresponding antibody (anti-CD11b-pe (Miltenyi Biotech, Germany) and anti-CD45-APC (Miltenyi Biotech, Germany) for microglia; anti-GLAST-APC (Miltenyi Biotech, Germany) for astrocyte) and prepared for flow cytometry assay by Attune NxT system (BD Biosciences, USA).

### Quantitative reverse transcription (RT) quantitative PCR

As previously described [23], total RNA from hippocampus was extracted by using TRIzol reagent (Invitrogen, Carlsbad, CA, USA) and reverse-transcribed under standard conditions using the PrimeScript^TM^ RT Reagent Kit (Takara, Tokyo, Japan). After that, the gene level was detected using the StepOnePlus^TM^ System (Applied Biosystems, Carlsbad, CA, USA) with SYBR Premix Ex Taq™ II (Takara) and the appropriate primers (Table S1) and Gapdh was used as the internal control.

### Adeno-associated virus (AAV) stereotaxic injection and CNO treatment

Designer receptors exclusively activated by designer drugs (DREADDs) application were performed as previous study described [24]. Chemogenetical virus AAV2/9-Gfap-hM4D(Gi)-mCherry-WPRE-pA (titer, 1.72 × 1013 vg/mL) was purchased from HANBIO Technology (Shanghai, China) and injected into the hippocampus of mice under anesthetic state which is induced by isoflurane (1.3%) for as much time as it takes and fixed on a stereotaxic apparatus (RWD, Shenzhen, China). The injected coordinate are AP, –2.00 mm; ML, ± 1.6 mm; DV, –2.0 mm; and AP, –3.00 mm, ML, ± 2.6 mm; and DV, –3.2 mm. To inhibit astrocyte, CNO (Clozapine N-oxide, 1 mg/kg/day, i.p., (MCE, Shanghai, China)) or saline (equal volume to CNO, i.p.) were injected into mice according to a former study [25].

### Microglia depletion

In order to evaluate the role of microglia on astrocytic activation, we eliminated microglia by feeding mice with PLX3397 according to previous study [26, 27]. Taking the patency (over 95% depletion efficiency) into consideration, we begin to provide PLX3397 (290mg/kg, Xietong Pharmaceutical Bio-engineering Co., JS, China) enriched chow for mice 7 days before the timepoint which microglia elimination is needed. Control mice was set by providing standard chow.

### LPS treatment

To activate the microglia after etomidate sedative application, we injected LPS (i.p.) into mice according to previous study [26]. In detail, LPS (Sigma-Aldrich, MO, USA) was dissolved by saline injection at a concentration of 0.1mg/ml and injected in mice at a dosage of 0.5mg/kg body weight. Control mice was set by injecting with saline.

### Statistical Analysis

Data were shown as mean ± standard errors of the mean (SEM) and analyzed by GraphPad Prism software (version 8.0.1, CA, USA). Two-tailed unpaired Student’s *t* test was used for comparing the difference between two groups, and two-way repeated-measures ANOVA with Bonferroni post hoc analysis was used for the Morris water maze test. Values with *p* < 0.05 were accepted as significant.

## Results

### Cognitive function and glial responses during the early pathological stage after etomidate application

**Fig. 1:**
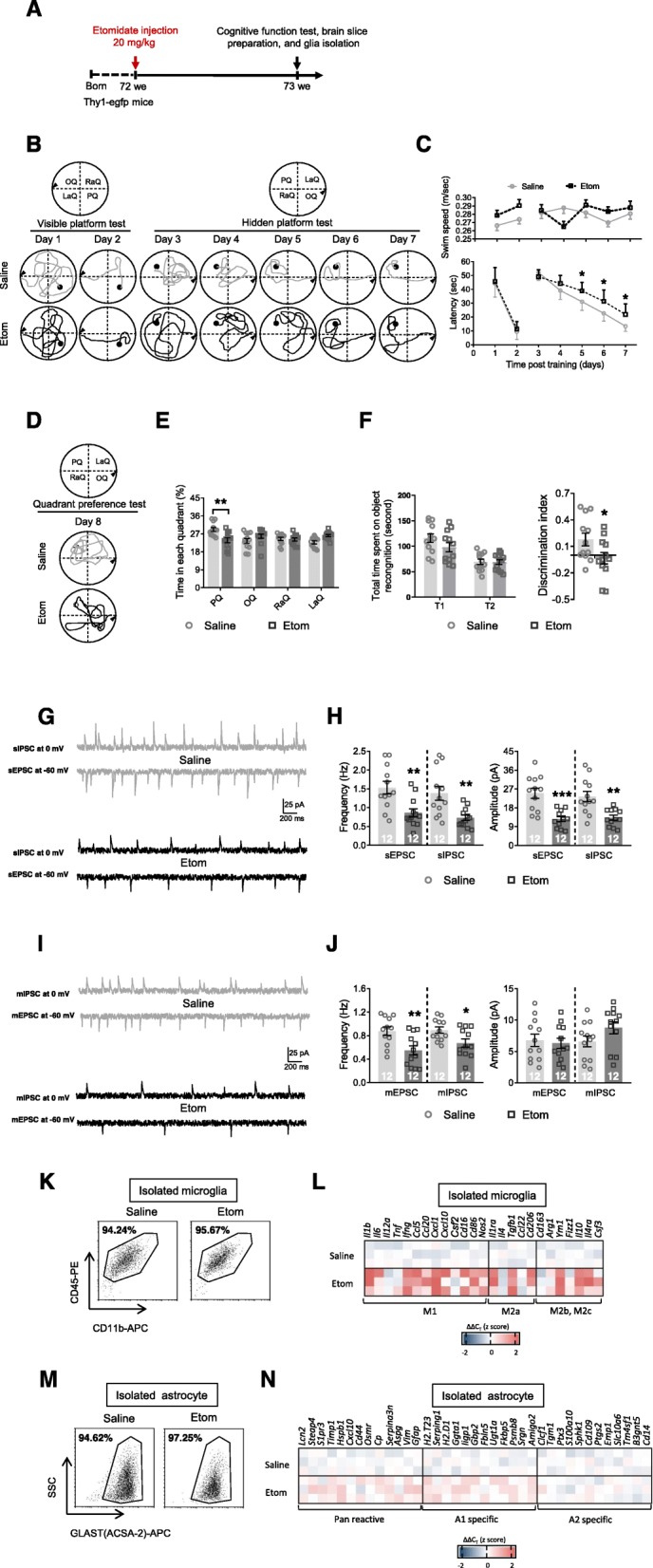
Etomidate anesthetic dosage application impairs cognition, depresses neuronal activity, and induced glial dysfunction in the early pathological stage (1 week post etomidate injection). **a** Graphic illustration of experimental procedure. During MWM test, we recorded swim path (**b**) and analyzed swim speed and latency to find platform (**c**) during visible and hidden platform training; (**d**) swim path and (**e**) time spent in each quadrant was investigated. **f** During NOR test, total time spent on object recognition and discrimination index were analyzed. Neuronal activities were detected by (**g** and **h**) sIPSC, sEPSC, (**i** and **j**) mIPSC, and mEPSC recording and analyzing (scale bar: 200 ms, 25 pA). Selected transcripts associated with microglial polarization (microglial phenotype of M1, M2a, and M2b,2c; **k** and **l**) and astrocyte activation (Pan-reactive, A1-specific, and A2-specific phenotype; M and N) were detected by qPCR. (*n* = 12 per group for behavioral and electrophysiological assay; *n* = 3 for qPCR assay; *p < 0.05, **p < 0.01, ***p < 0.001 compared with saline injected mice)

In this study, we generated a PND mouse model by administering etomidate at a dose of 20 mg/kg body weight to 18-month-old mice and evaluated cognitive function and glial responses 1 week later (Fig. 1a). The results showed that etomidate induced significant cognitive dysfunction, including spatial and non-spatial cognitive impairments. For example, escape latency on the hidden platform test was dramatically increased (Fig 1b, c), whereas time spent in the platform quadrant (i.e., the probe test; Fig. 1d, e) and the discrimination index (i.e., novel object recognition; Fig. 1f) were significantly decreased. In addition to the behavioral data, we also observed a suppression of neuronal activity in the hippocampus (i.e., reduced frequency of the spontaneous excitatory postsynaptic current [sEPSC], spontaneous inhibitory postsynaptic current [sIPSC], miniature [m]EPSC, and miniature [m]IPSC and the amplitude of the sEPSC and sIPSC; Fig. 1g–j). Furthermore, compared to control mice, data from isolated hippocampal microglia (Fig. 1k, l; Table S2) and astrocytes (Fig 1m, n; Table S2) of model mice showed that etomidate induced an non-specific microglial response during the early pathological stage, whereas a slight A1-like astrocyte expression profile (which is characterized by the expression of *H2.T23*, *Serping1*, *H2.D1*, *Ggta1*, *Ligp1*, *Gbp2*, *Fbln5*, *Ugt1a*, *Fkbp5*, *Psmb8*, *Srgn*, *Amigo2* [15]) was detected in isolated hippocampal astrocytes.

### Cognitive function and glial responses during the late pathological stage after etomidate application

**Fig. 2:**
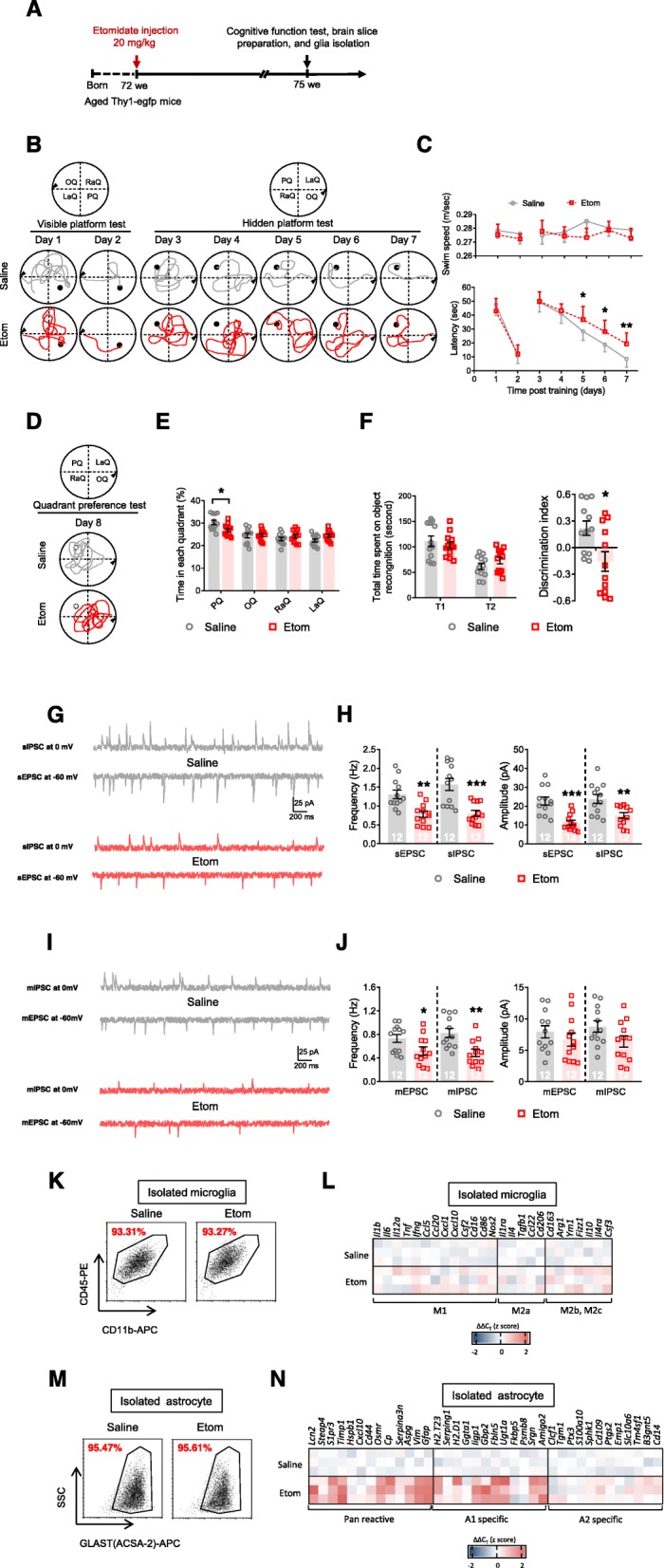
Etomidate anesthetic dosage application impairs cognition, depresses neuronal activity, and induced glial dysfunction in the late pathological stage (3 weeks post etomidate injection). **a** Graphic illustration of experimental procedure. During MWM test, we recorded swim path (**b**) and analyzed swim speed and latency to find platform (**c**) during visible and hidden platform training; (**d**) swim path and (**e**) time spent in each quadrant was investigated. **f** During NOR test, total time spent on object recognition and discrimination index were analyzed. Neuronal activities were detected by (**g** and **h**) sIPSC, sEPSC, (**i** and **j**) mIPSC, and mEPSC recording and analyzing (scale bar: 200 ms, 25 pA). Selected transcripts associated with microglial polarization (microglial phenotype of M1, M2a, and M2b,2c; **k** and **l**) and astrocyte activation (Pan-reactive, A1-specific, and A2-specific phenotype; **m** and **n**) were detected by qPCR. (*n* = 12 per group for behavioral and electrophysiological assay; *n* = 3 for qPCR assay; *p < 0.05, **p < 0.01, ***p < 0.001 compared with saline injected mice)

Etomidate administration results in long-term synaptic inhibition and cognitive dysfunction in older mice [24]. Therefore, we also assessed cognitive function and glial responses 3 weeks after the injections (Fig. 2a), in 18-month-old mice. We observed a notable increase in escape latency on the hidden platform test (Fig. 2b, c) and reduction in the time spent in the platform quadrant (i.e., the probe test; Fig. 2d, e) and in the discrimination index (i.e., novel object recognition; Fig. 2f). At the same time, we observed a dramatic inhibition of neuronal activity in the hippocampus (i.e., downregulated frequency of the sEPSC, sIPSC, mEPSC, and mIPSC and amplitude of the sEPSC and sIPSC; Fig. 2g–j). Subsequently, we assessed glial responses during the late pathological stage. In contrast to the previous data from the early pathological stage, we observed a slight non-specific microglial response (Fig. 2k, l; Table S3) and a clear A1-specific astrocyte response (Fig. 2m, n; Table S3).

### Depletion of microglia during the early pathological stage improves cognitive function during the late pathological stage by suppressing A1-specific astrocyte activation

**Fig. 3:**
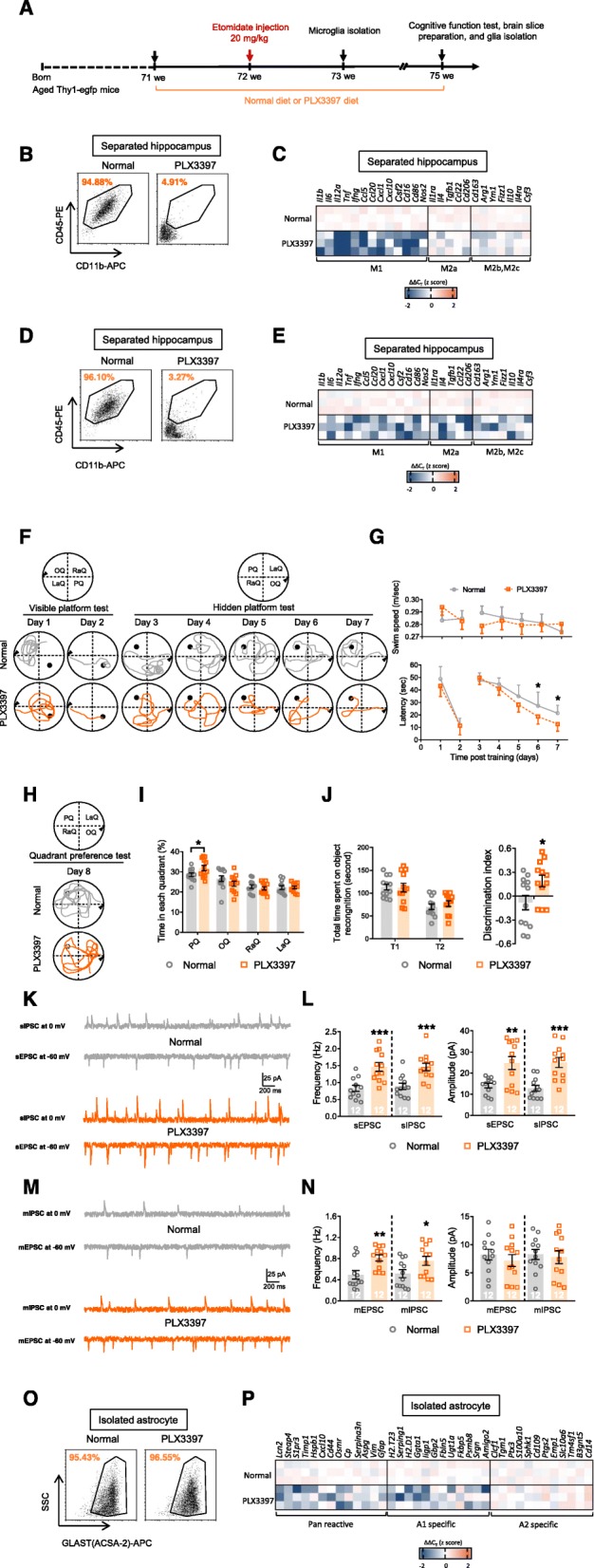
Microglial depletion before etomidate injection rescues cognitive, neuronal, and glial dysfunction in the late pathological stage (3 weeks post etomidate injection and 4 weeks post PLX3397 diet). **a** Graphic illustration of experimental procedure. Flow cytometry were applied to analyze the efficiency of microglial elimination and qPCR were applied to evaluate selected transcripts associated with microglial polarization (microglial phenotype of M1, M2a, and M2b,2c) in hippocampus after PLX3397 treatment at the early stage (1 week post etomidate injection and 2 weeks post PLX3397 diet; **b** and **c**) and the late stage (3 weeks post etomidate injection and 4 weeks post PLX3397 diet; **d** and **e**). During MWM test (3 weeks post etomidate injection and 4 weeks post PLX3397 diet), we recorded (**f**) swim path and (**g**) analyzed swim speed and latency to find platform during visible and hidden platform training; (**h**) swim path and (**i**) time spent in each quadrant was investigated. (**j**) During NOR test, total time spent on object recognition and discrimination index were analyzed. Neuronal activities were detected by (**k** and **l**) sIPSC, sEPSC, (**m** and **n**) mIPSC, and mEPSC recording and analyzing (scale bar: 200 ms, 25 pA). Selected transcripts associated with astrocyte activation (Pan-reactive, A1-specific, and A2-specific phenotype; **o** and **p**) were detected by qPCR. (*n* = 12 per group for behavioral and electrophysiological assay; *n* = 3 for qPCR assay; *p < 0.05, **p < 0.01, ***p < 0.001 compared with normal diet mice)

Considering these data together led us to speculate that the A1-specific astrocyte response is a downstream reaction that requires microglial activation. Therefore, to investigate the role of microglia in astrocyte function and long-term synaptic strength, we depleted microglia by PLX3397 treatment and investigated cognitive function and glial responses 3 weeks after etomidate injections (Fig. 3a), in aged (18-month-old) mice. As expected, compared to mice fed a normal diet, mice in the PLX3397 treatment group exhibited dramatically depleted microglia and depressed inflammatory responses in the hippocampus at both the early (Fig. 3b, c; Table S4) and late pathological stages (Fig. 3d, e; Table S4). Interestingly, microglial depletion significantly improved cognitive function, reducing escape latency in the hidden platform test (Fig. 3f, g) and increasing both time spent in the platform quadrant (i.e., the probe test; Fig. 3h, i) and the discrimination index (i.e., novel object recognition; Fig. 3j). It also upregulated hippocampal neuronal activities (i.e., increased the frequency of the sEPSC, sIPSC, mEPSC, and mIPSC and the amplitude of the sEPSC and sIPSC; Fig. 3k–n). Moreover, data from isolated hippocampal astrocytes showed that the elimination of microglia inhibited A1-astrocyte responses (Fig. 3o, p; Table S4).

### Microglia depletion during the late pathological stage had no significant effect on cognitive function and astrocyte responses

**Fig. 4:**
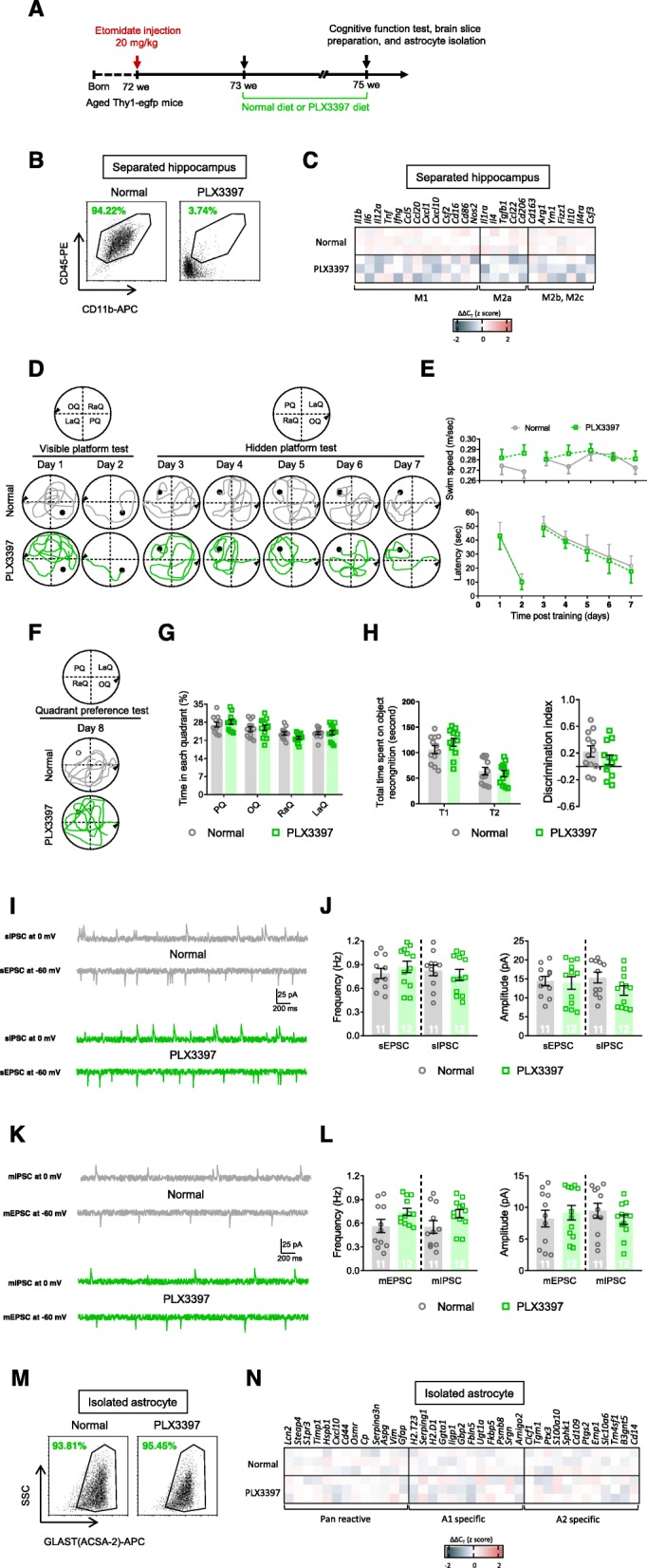
Microglial depletion at the late pathological stage of etomidate injection mice insignificantly influences cognitive, neuronal, and glial dysfunction in the late pathological stage (3 weeks post etomidate injection and 2 weeks post PLX3397 diet). **a** Graphic illustration of experimental procedure. **b** Flow cytometry were applied to analyze the efficiency of microglial elimination in hippocampus after PLX3397 treatment. **c** Selected transcripts associated with microglial polarization (microglial phenotype of M1, M2a, and M2b,2c) were detected by qPCR. During MWM test, we recorded swim path (**d**) and analyzed swim speed and latency to find platform (**e**) during visible and hidden platform training; (**f**) swim path and (**g**) time spent in each quadrant was investigated. **h** During NOR test, total time spent on object recognition and discrimination index were analyzed. Neuronal activities were detected by (**i** and **j**) sIPSC, sEPSC, (**k** and **l**) mIPSC, and mEPSC recording and analyzing (scale bar: 200 ms, 25 pA). Selected transcripts associated with astrocyte activation (Pan-reactive, A1-specific, and A2-specific phenotype; **m** and **n**) were detected by qPCR. (*n* = 11-12 per group for behavioral and electrophysiological assay; *n* = 3 for qPCR assay; *p < 0.05, **p < 0.01, ***p < 0.001 compared with normal diet mice)

We assessed whether A1-astrocyte responses were triggered by transient microglial activation during the early pathological stage or required a constant inflammatory environment. Therefore, we depleted microglia by PLX3397 treatment during the late pathological stage and investigated cognitive function and glial responses at 3 weeks after etomidate injection (Fig. 4a), in 18-month-old mice. The data showed that more than 95% of the microglia were eliminated (Fig. 4b), and inflammatory responses were dramatically suppressed (Fig. 4c; Table S5) 3 weeks after etomidate administration. However, the cognitive function test parameters, including escape latency on the hidden platform test (Fig. 4d, e), time spent in the platform quadrant (i.e., the probe test; Fig. 4f, g), and the discrimination index (i.e., novel object recognition; Fig. 4h), as well as neuronal activity (i.e., the frequency of the sEPSC, sIPSC, mEPSC, and mIPSC and the amplitude of the sEPSC and sIPSC, mEPSC, and mIPSC; Fig. 4i–l) were not significantly altered. In addition, astrocyte responses were unaffected (Fig. 4m, n; Table S5).

### Activating microglia before etomidate sedative administration induces significant cognitive dysfunction

**Fig. 5:**
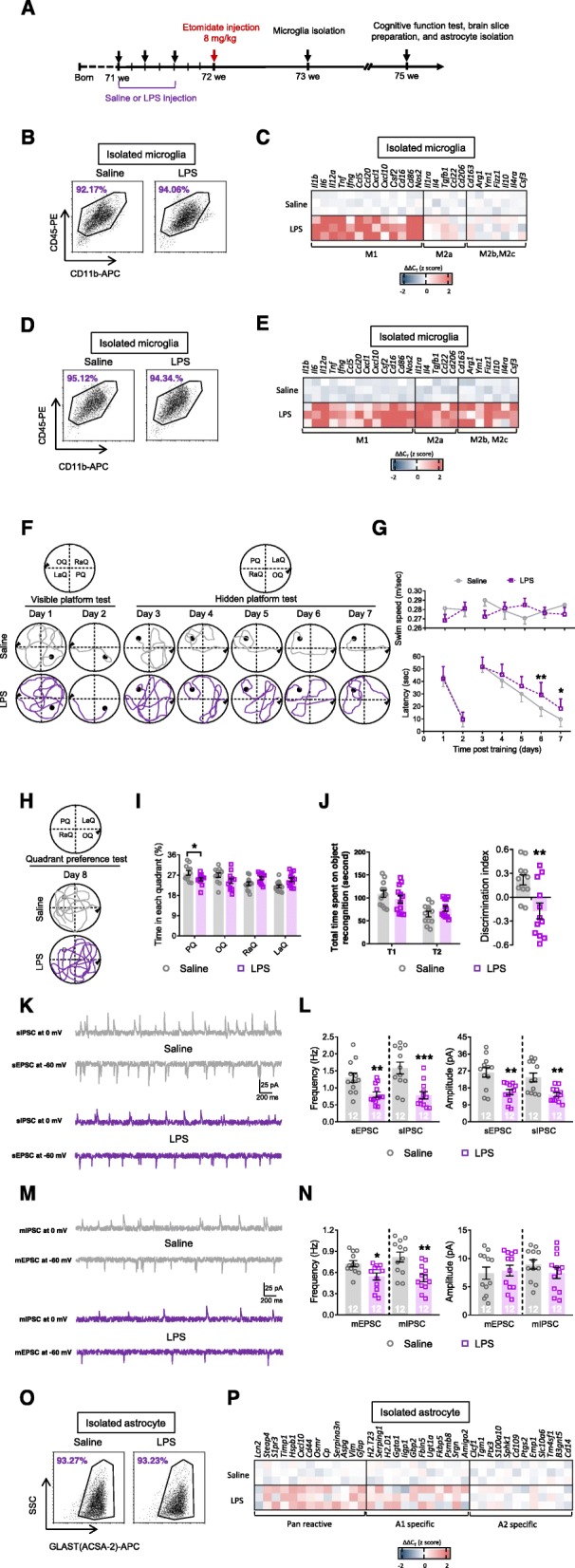
LPS treatment before etomidate sedative injection impairs cognitive, neuronal, and glial function in the late pathological stage (3 weeks post etomidate sedative injection and 4 weeks post LPS injection). **a** Graphic illustration of experimental procedure. Flow cytometry were applied to analyze the efficiency of microglial activation and qPCR was applied to evaluate the selected transcripts associated with microglial polarization (microglial phenotype of M1, M2a, and M2b,2c) in hippocampus after LPS treatment at the early stage (1 week post etomidate sedative injection and 2 weeks post LPS injection; **b** and **c**) and late stage (3 weeks post etomidate sedative injection and 4 weeks post LPS injection; **d** and **e**). During MWM test (3 weeks post etomidate sedative injection and 4 weeks post LPS injection), we recorded (**f**) swim path and (**g**) analyzed swim speed and latency to find platform during visible and hidden platform training; **(h**) swim path and (**i**) time spent in each quadrant was investigated. **j** During NOR test, total time spent on object recognition and discrimination index were analyzed. Neuronal activities were detected by (**k** and **l**) sIPSC, sEPSC, (**m** and **n**) mIPSC, and mEPSC recording and analyzing (scale bar: 200 ms, 25 pA). Selected transcripts on regulation astrocyte activation (Pan-reactive, A1-specific, and A2-specific phenotype; **o** and **p**) were detected by qPCR. (*n* = 12 per group for behavioral and electrophysiological assay; *n* = 3 for qPCR assay; *p < 0.05, **p < 0.01, ***p < 0.001 compared with saline injected mice)

Next, we assessed whether activating microglia before etomidate administration triggered an A1-astrocyte response and further negative effects. We administered an intraperitoneal LPS injection to mice before the etomidate sedative injections and assessed cognitive function and astrocyte responses during the late pathological stage (Fig. 5a), in 18-month-old mice. Notably, the LPS injections promoted multiple pro-inflammatory transcripts in isolated microglia at the early pathological stage (Fig. 5b, c; Table S6) and concomitantly upregulated several pro-inflammatory and anti-inflammatory transcripts in isolated microglia at the late pathological stages (Fig. 5d, e; Table S6), leading to impaired cognitive function, as indicated by increased escape latency in the hidden platform test (Fig. 5f, g) and decreases in both time spent in the platform quadrant (i.e., the probe test; Fig. 5h, i) and discrimination index scores (i.e., novel object recognition; Fig. 5j). In addition, neuronal activities in the hippocampus were dramatically downregulated (i.e., reductions in the frequency of the sEPSC, sIPSC, mEPSC, and mIPSC and in the amplitude of the sEPSC and sIPSC; Fig. 5k–n). Furthermore, a slight A1-specific astrocyte response was observed (Fig. 5o, p; Table S6).

### Inhibiting astrocytes reversed the microglial-activation-induced cognitive deficiencies observed after etomidate application

**Fig. 6:**
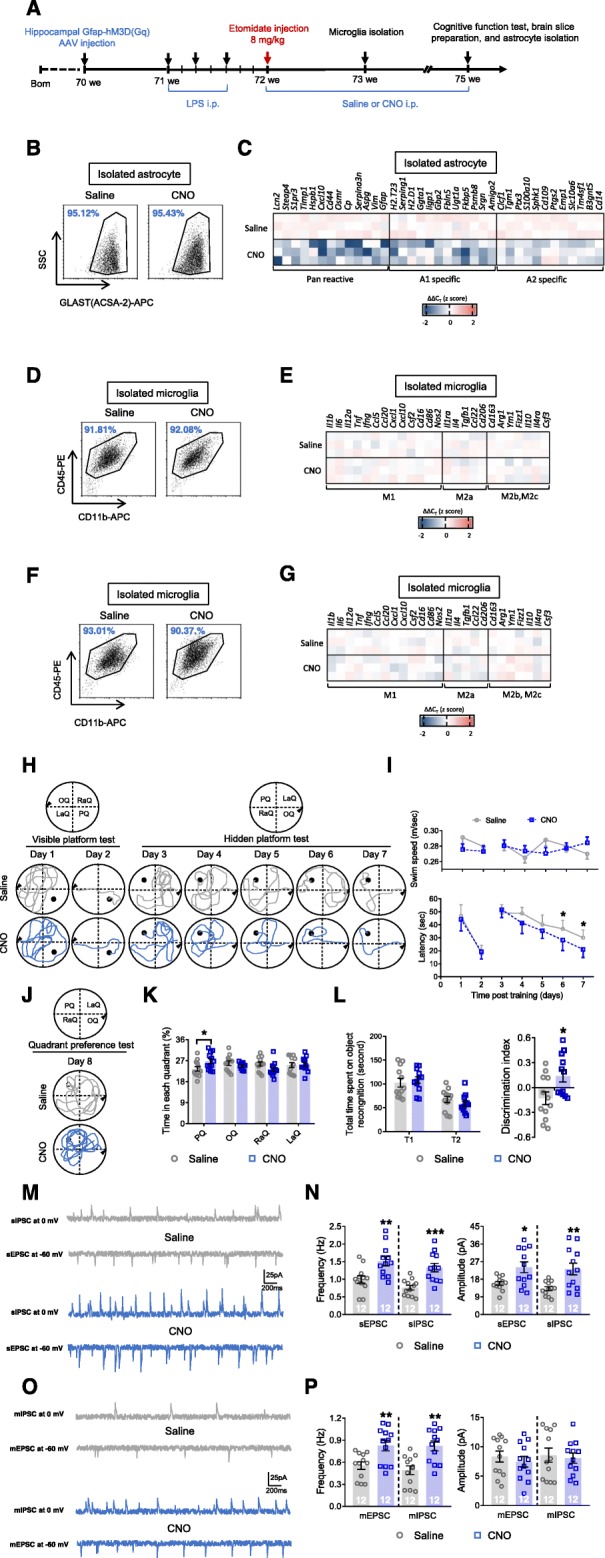
Astrocytic inhibition reverses LPS treatment induced neuronal and glial dysfunction in the late pathological stage in mice after etomidate sedative injection (3 weeks post etomidate sedative injection). (**a**) Graphic illustration of experimental procedure. Selected transcripts on regulation astrocytic activation (Pan-reactive, A1-specific, and A2-specific phenotype; **b** and **c**) were detected by qPCR. Flow cytometry were applied to analyze the cell purity of microglia after MACS isolation and qPCR were applied to evaluate selected transcripts associated with microglial polarization (microglial phenotype of M1, M2a, and M2b,2c) were detected by qPCR at the early stage (1 week post etomidate sedative injection; **d** and **e**) and late stage (3 week post etomidate sedative injection; **f** and **g**). During MWM test (3 weeks post etomidate sedative injection), we recorded (**h**) swim path and (**i**) analyzed swim speed and latency to find platform during visible and hidden platform training; (**j**) swim path and (**k**) time spent in each quadrant was investigated. **l** During NOR test, total time spent on object recognition and discrimination index were analyzed. Neuronal activities were detected by (**m** and **n**) sIPSC, sEPSC, (**o** and **p**) mIPSC, and mEPSC recording and analyzing (scale bar: 200 ms, 25 pA). (*n* = 12 per group for behavioral and electrophysiological assay; *n* = 3 for qPCR assay; *p < 0.05, **p < 0.01, ***p < 0.001 compared with saline injected mice)

Astrocyte dysfunction causes long-term synaptic inhibition and cognitive impairments [24]. However, it is unclear whether microglial-activation-induced inflammatory responses can induce these impairments without triggering A1-specific astrocyte responses. Therefore, in addition to activating microglia by LPS injection after etomidate administration, we also inhibited astrocyte activation using designer receptors exclusively activated by designer drugs (Fig. 6a), in 18-month-old mice. The results suggested that clozapine-N-oxide treatment inhibited astrocyte activation during the late pathological stage (Fig. 6b, c; Table S7) but did not change microglial responses to LPS administration in the early (Fig. 6d, e; Table S7) and late pathological stages (Fig. 6f, g; Table S7). In contrast, inhibiting astrocytes reversed microglial-activation-induced cognitive decline during the late pathological stage, reducing escape latency on the hidden platform test (Fig. 6h, i), increasing the time spent in the platform quadrant (i.e., the probe test; Fig. 6j, k), the discrimination index (i.e., novel object recognition; Fig. 6l), and synaptic inhibition (i.e., upregulating the frequency of the sEPSC, sIPSC, mEPSC, and mIPSC, as well as the amplitude of the sEPSC and sIPSC; Fig. 6m–p).

## Discussion

The current data suggest that both the essential and the undesired effects of anesthesia can be attributed to synaptic inhibition. Postsynaptic GABAA-R channels trigger fast and transient phasic inhibitory currents that produce amnesia, unconsciousness, and immobility, whereas extrasynaptic GABAA-R channels induce slow and persistent tonic inhibitory currents that produce undesirable effects, such as delirium and cognitive deficiencies [10]. However, many unanswered questions remain, such as (i) why does extrasynaptic GABAA-R induced long-term synaptic inhibition require astrocyte activation [14]; (ii) why are etomidate-generated GABAA-R tonic inhibitory currents alone insufficient to cause amnesia, and what other components are involved [28]; (iii) what roles do microglial and astrocyte activation play in regulating synaptic inhibition during and after anesthesia [24, 29]; (iv) is there crosstalk between the microglial and astrocyte pathways; and (v) can microglial or astrocytic inhibition alone rescue long-term synaptic inhibition and cognitive dysfunction?

### Non-specific microglial (early) and A1-specific astrocytic (late) responses were processed after etomidate administration in a fixed order

Accumulating evidence has shown that glial responses promote pathogenesis and cognitive dysfunction after general anesthetics are administered. For example, cytokines (especially HMGB1, NFκB, and TNFα [30]) secreted from circulating or fixed mononuclear cells regulated microglial feedback reactions [31], upregulating MCP-1 expression and inflammatory cascade signaling pathways and triggering neuroinflammation in the hippocampus [32]. In addition, microglial elimination or inhibition suppressed hippocampal inflammatory responses and reduced the levels of cytokines such as IL-6, IL-1β, and TNFα following anesthesia and other pathological conditions [17, 33, 34]. Moreover, the level of inositol 1,4,5-triphosphate type 2 receptor-dependent calcium was reduced in astrocytes, even after a sedative dose of anesthetic [35], and long-term inhibition of astrocytes is consistent with synaptic suppression and cognitive deficiencies [24]. Interestingly, in this study, non-specific microglial activation occurred during the early pathological stage, together with synaptic inhibition and cognitive dysfunction, which were slightly alleviated during the late pathological stage, in an aged (18-month-old) PND mice model. In contrast to microglial activation, astrocyte expression property was not significantly altered during the early pathological stage but was stimulated in an A1-specific manner during the late pathological stage. These data suggest that (i) non-specific microglial activation may enable multiple cytokines to create an inflammatory environment, (ii) non-specific microglial responses or an inflammatory environment may be required to trigger the A1-specific astrocytic response, and (iii) A1-specific astrocytes mediate long-term synaptic inhibition and cognitive decline.

### Etomidate-induced long-term synaptic inhibition is rescued after microglial depletion before the early pathological stage but not during the late pathological stage

To evaluate the role of microglia in cognitive dysfunction after etomidate administration, we depleted microglia before anesthesia and found that this improved cognitive function and upregulated neuronal activity. This observation, together with previous results showing that inhibiting microglia activation [18] or eliminating microglia [17] dramatically enhanced cognitive function, highlights the importance of microglial activation and neuroinflammation in PND pathogenesis. However, the mechanism by which microglia mediate synaptic suppression and cognitive impairments remains unclear.

Under physiological and pathological conditions, microglia mediate synaptic suppression directly and trigger synaptic inhibition via neuroinflammation indirectly [36–38]. To determine whether microglial-induced synaptic elimination was a key factor in regulating long-term synaptic inhibition and cognitive deficiencies, we compared the cognitive function, neuronal activities, and astrocytic responses of PND mice that were treated with PLX3397 before anesthesia (depleting microglia throughout pathological progression) and PND mice (18-month-old) that were treated with PLX3397 1 week after anesthesia (depleting microglia during the late pathological stage). In contrary to the data obtained by depleting microglia throughout pathological progression, no significant difference were observed between these groups by depleting microglia during the late pathological stage, suggesting that microglial activation may worsen the situation at the late pathological stage, but was not the only factor regulating long-term synaptic inhibition and cognitive impairment. Furthermore, astrocyte responses were not significantly affected by depleting microglia during the late pathological stage. This suggests three possibilities: (i) non-specific microglial responses during the late pathological stage of PND have no effect on long-term synaptic inhibition and cognitive dysfunction; (ii) non-specific microglial responses during the late pathological stage of PND actuate synaptic inhibition, which requires A1-specific astrocytic responses; or (iii) non-specific microglial activation during the late pathological stage of PND induces long-term synaptic inhibition and cognitive dysfunction indirectly and in an A1-specific astrocyte-response-independent manner.

### Non-specific microglial activation is necessary to inhibit long-term synaptic plasticity and cognitive function in PND

Our understanding of microglial functions depends on the application of novel cutting-edge technologies (e.g., two-photon imaging, transgenic models, and whole-genome transcriptomics). These functions include dynamic and context-dependent pro-inflammatory and anti-inflammatory gene expression, morphological changes, immune monitoring, synaptic refinement, apoptosis, and cytokine-secretion-mediated neuron survival [39]. Although microglial gene responses may not correspond to discrete cell populations, the use of the designations M1 and M2 is helpful in describing specific patterns of gene expression related to microglial activation states. Therefore, the pro-inflammatory state, designated the M1-phenotype, is characterized by the expression of IL-1β, IL-6, IL-12p70, TNF, IFNγ, CCL5, CCL20, CXCL1, CXCL10, GM-CSF, CD16, CD86, and iNOS. The anti-inflammatory state is designated the M2-phenotype and is further subdivided into the M2a-phenotype (which expresses IL-1Ra, IL-4, TGFβ, CCL22, and CD206) and the M2b- and M2c-phenotypes (which express CD163, Arg1, Ym1, FIZZ1, IL-10, IL-4Rα, and G-CSF) [40]. We observed non-specific microglial activation after etomidate administration and tested whether etomidate-triggered non-specific activation was important for inducing long-term synaptic inhibition and cognitive dysfunction, after anesthesia in aged (18-month-old) mice.

A sedative dose of etomidate is reportedly [14] sufficient to generate phasic synaptic inhibition and short-term memory impairment after 72 h, but fails to produce persistent long-term synaptic inhibition and cognitive impairment after 1 week. Therefore, due to this difference between the early and late stage effects of etomidate on synaptic plasticity and cognition, we investigated the role of microglial activation on inducing long-term synaptic deficiencies by administering LPS treatment before anesthesia [26, 41]. Interestingly, LPS-mediated microglial activation was sufficient to impair synaptic and cognitive function during the late pathological stage, after anesthesia in aged (18-month-old) mice. In addition, microglial depletion before etomidate administration rescued long-term synaptic inhibition, but microglial depletion during the late pathological stage failed to reverse synaptic dysfunction. These data suggest that LPS-mediated microglial activation is an essential pathological component that aggravates synaptic impairment. Furthermore, LPS-mediated microglial activation also stimulates A1-specific astrocyte responses during the late pathological stage, but whether these responses are required for long-term synaptic inhibition remains unclear.

### A1-specific astrocyte activation, stimulated by microglial activation, plays a critical role in long-term synaptic inhibition and cognitive deficiencies in PND

Previous studies have emphasized the vital role of astrocyte dysfunction in causing cognitive dysfunction [24, 42]. Dysfunctional astrocytes have been detected during the late pathological stage of PND. Two recent studies have highlighted the neurotoxic A1-specific astrocyte response, which is downstream of microglial activation [15, 16]. Therefore, we investigated whether there was an association between the activation of microglia and A1-specific astrocytes in PND. Suppressing astrocyte activation during the late pathological stage prevented LPS from producing synaptic inhibition and cognitive deficiencies. This confirmed that A1-specific astrocyte responses linked early pathological microglial responses with long-term synaptic inhibition and cognitive deficiencies. However, the mechanisms of these pathways are unclear, and further studies are needed to reveal (i) the factors responsible for the initiation of pro-inflammatory microglial activation and neuroinflammation, (ii) whether there is a specific target for inhibiting pro-inflammatory microglial activation, (iii) how the microglia trigger A1-specific astrocytic responses, (iv) whether a particular factor can be targeted to prevent microglial activation at the early pathological stage, and (v) whether a supportive environment can be built to maintain astrocyte function. Additionally, one limitation to our study is that all experiments were carried out in male mice to eliminate the unexpected and ambiguous impact generated by gender [43, 44], thus future studies with female mice might be helpful in exploring such differences.

## Conclusions

In conclusion, our results show that microglial activation during the early pathological stage of PND creates an inflammatory environment and stimulates A1-specific astrocyte responses during the late pathological stage, in the aged (18-month-old) mice. This eventually induces persistent synaptic inhibition and cognitive deficiencies. These results enhance our understanding of PND pathogenesis and highlight the importance of microglia–astrocyte crosstalk during the early pathological stage.

## Supplementary information

**Additional file 1.** .

**Additional file 2.** .

**Additional file 3.** .

**Additional file 4.** .

**Additional file 5.** .

**Additional file 6.** .

**Additional file 7.** .

## Data Availability

The datasets used and/or analyzed details during the current study are available from the corresponding author on reasonable request.

## Abbreviations

aCSF: artificial cerebrospinal fluid
CCL: CCR-like protein
CXCL: Chemokine (C-X-C motif) ligand
ED: Effective dose
GABA: γ-aminobutyric acid
GABAA-R: GABA type A receptor
GM-CSF: Granulocyte-macrophage colony-stimulating factor
IFN: Interferon
IL: Interleukin
iNOS: Inducible nitric oxide synthase
MACS: Magnetic-activated cell-sorting
mEPSC: miniature excitatory postsynaptic currents
mIPSC: miniature inhibitory postsynaptic currents
PND: Perioperative neurocognitive disorders
POCD: Postoperative cognitive dysfunction
sEPSC: spontaneous excitatory postsynaptic currents
sIPSC: spontaneous inhibitory postsynaptic currents
TGF: Transforming growth factor
TNF: Tumor necrosis factor
