# Supplementary material for: Hippocampal microglial activation triggers a neurotoxic-specific astrocyte response and mediates etomidate-induced long-term synaptic inhibition
Source: J Neuroinflammation. 2020 Apr 7;17:109. doi: 10.1186/s12974-020-01799-0 (PMC7140340; doi:10.1186/s12974-020-01799-0)
Supplement: Supplementary file 1 — Additional file 1. . [file 12974_2020_1799_MOESM1_ESM.docx]

Table S1. Primers used in present study.

| \| **Gene** \| **Forward primer (5'-3')** \| **Reverse primer (5'-3')** \| **Amplicon size** \| \| --- \| --- \| --- \| --- \| \| *Amigo2* \| GTTCGCCACAACAACATCAC \| GTTTCTGCAAGTGGGAGAGC \| 211 \| \| *Arg1* \| CTCCAAGCCAAAGTCCTTAGAG \| GGAGCTGTCATTAGGGACATCA \| 184 \| \| *Aspg* \| CAGGTGCCCAGGTTCCTATC \| GTCCACCTTGGTTGTCCGAT \| 152 \| \| *B3gnt5* \| TGCTCCTGGATGAAAGGTCC \| ACATGCTTGATCCGTGTGGT \| 161 \| \| *Ccl20* \| GCCTCTCGTACATACAGACGC \| CCAGTTCTGCTTTGGATCAGC \| 146 \| \| *Ccl22* \| ATGCGCGTCCATTACCTGTG \| TCAACGGTCCAATCATTTGCT \| 86 \| \| *Ccl5* \| TTTGCCTACCTCTCCCTCG \| CGACTGCAAGATTGGAGCACT \| 85 \| \| *Cd109* \| GTCGCTCACAGGTACCTCAA \| CTGTGAAGTTGAGCGTTGGC \| 116 \| \| *Cd14* \| TCAGAATCTACCGACCATGAAGC \| GGACACTTTCCTCGTCCTGG \| 119 \| \| *Cd16* \| AATGCACACTCTGGAAGCCAA \| CACTCTGCCTGTCTGCAAAAG \| 79 \| \| *Cd163* \| GGTGGACACAGAATGGTTCTTC \| CCAGGAGCGTTAGTGACAGC \| 128 \| \| *Cd206* \| CTCTGTTCAGCTATTGGACGC \| TGGCACTCCCAAACATAATTTGA \| 190 \| \| *Cd44* \| TCAGGATAGCCCCACAACAAC \| GACTCCGTACCAGGCATCTTC \| 159 \| \| *Cd86* \| TCAATGGGACTGCATATCTGCC \| GCCAAAATACTACCAGCTCACT \| 78 \| \| *Clcf1* \| GACTCGTGGGGGATGTTAGC \| CCCCAGGTAGTTCAGGTAGGT \| 180 \| \| *Cp* \| GATGTTTCCCCAAACGCCTG \| GTAGCTCTGAGACGATGCTTGA \| 118 \| \| *Csf2* \| GGCCTTGGAAGCATGTAGAGG \| GGAGAACTCGTTAGAGACGACTT \| 104 \| \| *Csf3* \| ATGGCTCAACTTTCTGCCCAG \| CTGACAGTGACCAGGGGAAC \| 110 \| \| *Cxcl1* \| CTGGGATTCACCTCAAGAACATC \| CAGGGTCAAGGCAAGCCTC \| 117 \| \| *Cxcl10* \| TGCAAGTCTATCCTGTCCGC \| ACGGAGCTCTTTTTGACCTTC \| 140 \| \| *Cxcl10* \| CCAAGTGCTGCCGTCATTTTC \| GGCTCGCAGGGATGATTTCAA \| 157 \| \| *Emp1* \| ACCATTGCCAACGTCTGGAT \| TGGAACACGAAGACCACGAG \| 188 \| \| *Fbln5* \| AGGGGGTTAAGCGAAACCAG \| GTGAGTATCCTTTTAATCCTGGCA \| 198 \| \| *Fizz1* \| CCAATCCAGCTAACTATCCCTCC \| ACCCAGTAGCAGTCATCCCA \| 108 \| \| *Fkbp5* \| TGCAGTGTCGGCAGTTGTAT \| GGGTCGCCCAAGTTAGAACA \| 112 \| \| *Gapdh* \| AGGTCGGTGTGAACGGATTTG \| GGGGTCGTTGATGGCAACA \| 95 \| \| *Gbp2* \| TAAAGGTCCGAGGCCCAAAC \| AACATATGTGGCTGGGCGAA \| 192 \| \| *Gfap* \| AACCGCATCACCATTCCTGT \| TCCTTAATGACCTCGCCATCC \| 146 \| \| *Ggta1* \| TCTCAGGATCTGGGAGTTGGA \| GAGTTCTATGGAGCTCCCGC \| 84 \| \| *H2.D1* \| ATGGAACCTTCCAGAAGTGGG \| GAAGTAAGTTGGAGTCGGTGGA \| 144 \| \| *H2.T23* \| ATTGGAGCTGTTGTGAGGAGG \| CCACGAGGCAACTGTCTTTTC \| 130 \| \| *Hspb1* \| GAGATCACTGGCAAGCACGA \| ATTGTGTGACTGCTTTGGGC \| 172 \| \| *Ifng* \| ATGAACGCTACACACTGCATC \| CCATCCTTTTGCCAGTTCCTC \| 182 \| \| *Iigp1* \| ATTTGGCTCGAAGCCTTTGC \| ACGGCATTTGCCAGTCCTTA \| 169 \| \| *Il10* \| CTTACTGACTGGCATGAGGATCA \| GCAGCTCTAGGAGCATGTGG \| 101 \| \| *Il12a* \| AGACATCACACGGGACCAAAC \| CCAGGCAACTCTCGTTCTTGT \| 77 \| \| *Il1b* \| GAAATGCCACCTTTTGACAGTG \| TGGATGCTCTCATCAGGACAG \| 116 \| \| *Il1ra* \| GCTCATTGCTGGGTACTTACAA \| CCAGACTTGGCACAAGACAGG \| 132 \| \| *Il4* \| GGTCTCAACCCCCAGCTAGT \| GCCGATGATCTCTCTCAAGTGAT \| 102 \| \| *Il4ra* \| ACACTACAGGCTGATGTTCTTCG \| TGGACCGGCCTATTCATTTCC \| 108 \| \| *Il6* \| CTGCAAGAGACTTCCATCCAG \| AGTGGTATAGACAGGTCTGTTGG \| 131 \| \| *Lcn2* \| CCGACACTGACTACGACCAG \| AATGCATTGGTCGGTGGGAA \| 197 \| \| *Nos2* \| GTTCTCAGCCCAACAATACAAGA \| GTGGACGGGTCGATGTCAC \| 127 \| \| *Osmr* \| GTCATTCTGGACATGAAGAGGT \| AATCACAGCGTTGGGTCTGA \| 144 \| \| *Psmb8* \| TATCTGCGGAATGGGGAACG \| AAAGTCCCGGTCCCTTCTTG \| 136 \| \| *Ptgs2* \| CTCAGCCATGCAGCAAATCC \| GGGTGGGCTTCAGCAGTAAT \| 172 \| \| *Ptx3* \| CATCCCGTTCAGGCTTTGGA \| CACAGGGAAAGAAGCGAGGT \| 104 \| \| *S100a10* \| GAAAGGGAGTTCCCTGGGTT \| CCCACTTTTCCATCTCGGCA \| 98 \| \| *S1pr3* \| CTTGCAGAACGAGAGCCTGT \| CCTCAACAGTCCACGAGAGG \| 70 \| \| *Serpina3n* \| GTCTTTCAGGTGGTCCACAAGG \| GCCAATCACAGCATAGAAGCG \| 297 \| \| *Serping1* \| TGGCTCAGAGGCTAACTGGC \| GAATCTGAGAAGGCTCTATCCCCA \| 122 \| \| *Slc10a6* \| TCCATAGAGACCGGAGCACA \| ATGCCTGATATGCTGCGACA \| 157 \| \| *Sphk1* \| AAAGCGAGACCCTGTTCCAG \| CAGTCTGCTGGTTGCATAGC \| 231 \| \| *Srgn* \| GTTCAAGGTTATCCTGCTCGGA \| AAACAGGATCGGTCATCGGG \| 151 \| \| *Steap4* \| CAAACGCCGAGTACCTTGCT \| CAGACAAACACCTGCCGACT \| 121 \| \| *Tgfb1* \| CCACCTGCAAGACCATCGAC \| CTGGCGAGCCTTAGTTTGGAC \| 91 \| \| *Tgm1* \| AGACCCAATTTTCCTGGGGC \| AGCGAGGACCTTCCATTGTG \| 100 \| \| *Timp1* \| CGCTAGAGCAGATACCACGA \| CCAGGTCCGAGTTGCAGAAA \| 140 \| \| *Tm4sf1* \| CTGAGGGACAGTACCTTCTGGATTC \| GGCTAGGCCTCAACACAGTTA \| 225 \| \| *Tnf* \| CAGGCGGTGCCTATGTCTC \| CGATCACCCCGAAGTTCAGTAG \| 89 \| \| *Ugt1a* \| GGAAGCTGTTAGTGATCCCC \| TGCTATGACCACCACTTCGT \| 101 \| \| *Vim* \| GAGGAGATGAGGGAGTTGCG \| CTGCAATTTTTCTCGCAGCC \| 117 \| \| *Ym1* \| CAGGTCTGGCAATTCTTCTGAA \| GTCTTGCTCATGTGTGTAAGTGA \| 197 \| |  |  |  |
| --- | --- | --- | --- | --- | --- | --- | --- | --- | --- | --- | --- | --- | --- | --- | --- | --- | --- | --- | --- | --- | --- | --- | --- | --- | --- | --- | --- | --- | --- | --- | --- | --- | --- | --- | --- | --- | --- | --- | --- | --- | --- | --- | --- | --- | --- | --- | --- | --- | --- | --- | --- | --- | --- | --- | --- | --- | --- | --- | --- | --- | --- | --- | --- | --- | --- | --- | --- | --- | --- | --- | --- | --- | --- | --- | --- | --- | --- | --- | --- | --- | --- | --- | --- | --- | --- | --- | --- | --- | --- | --- | --- | --- | --- | --- | --- | --- | --- | --- | --- | --- | --- | --- | --- | --- | --- | --- | --- | --- | --- | --- | --- | --- | --- | --- | --- | --- | --- | --- | --- | --- | --- | --- | --- | --- | --- | --- | --- | --- | --- | --- | --- | --- | --- | --- | --- | --- | --- | --- | --- | --- | --- | --- | --- | --- | --- | --- | --- | --- | --- | --- | --- | --- | --- | --- | --- | --- | --- | --- | --- | --- | --- | --- | --- | --- | --- | --- | --- | --- | --- | --- | --- | --- | --- | --- | --- | --- | --- | --- | --- | --- | --- | --- | --- | --- | --- | --- | --- | --- | --- | --- | --- | --- | --- | --- | --- | --- | --- | --- | --- | --- | --- | --- | --- | --- | --- | --- | --- | --- | --- | --- | --- | --- | --- | --- | --- | --- | --- | --- | --- | --- | --- | --- | --- | --- | --- | --- | --- | --- | --- | --- | --- | --- | --- | --- | --- | --- | --- | --- | --- | --- | --- | --- | --- | --- | --- | --- | --- | --- | --- | --- | --- | --- | --- | --- | --- | --- | --- | --- | --- |
|  |  |  |  |
|  |  |  |  |
|  |  |  |  |
